# A Meta-Analysis of Association between Methylenetetrahydrofolate Reductase Gene (MTHFR) 677C/T Polymorphism and Diabetic Retinopathy

**DOI:** 10.3390/ijerph13080806

**Published:** 2016-08-10

**Authors:** Shasha Luo, Furu Wang, Chao Shi, Zhifeng Wu

**Affiliations:** 1 Department of Ophthalmology, Nanjing Medical University Affiliated Wuxi Second Hospital, 68 Zhongshan Road, Wuxi 214002, China; luoshasha403@163.com; 2 Jiangsu Provincial Center for Disease Prevention and Control, 172 Jiangsu Road, Nanjing 210029, China; wangfuru1983@126.com; 3 Wuxi Center for Disease Control and Prevention, 499 Jincheng Road, Wuxi 214023, China

**Keywords:** *MTHFR* 677C/T, polymorphism, diabetic retinopathy, DM type, ethnicity

## Abstract

*Aims*: To shed light on the conflicting findings of the association between the methylenetetrahydrofolate reductase gene (*MTHFR*) 677C/T polymorphism and the risk of diabetic retinopathy (DR), a meta-analysis was conducted. *Methods*: A predefined search was performed on 1747 DR cases and 3146 controls from 18 published studies by searching electronic databases and reference lists of relevant articles. A random-effects or fixed-effects model was used to estimate the sizes of overall and stratification effects of the *MTHFR* 677C/T polymorphism on the risk of DR, as appropriate. *Results*: Risks were evaluated by odds ratios (OR) with 95% confidence intervals (95% CI). We found a significant association between the *MTHFR* 677C/T polymorphism and the risk of DR for each genetic model (recessive model: OR = 1.67; 95% CI: 1.19–2.40 and dominant model: OR = 1.71; 95% CI: 1.28–2.28; respectively). In stratified analysis; we further found that the Asian group with both types of diabetes mellitus (DM) showed a significant association with genetic models (recessive model: OR = 2.16; 95% CI: 1.75–2.60 and dominant model: OR = 1.98; 95% CI: 1.42–2.76; respectively). *Conclusions*: Our study suggested that the *MTHFR* 677C/T polymorphism may contribute to DR development, especially in Asian populations. Prospective and additional genome-wide association studies (GWAS) are needed to clarify the real role of the *MTHFR* gene in determining susceptibility to DR.

## 1. Introduction

Diabetic retinopathy (DR) is the leading cause of vision loss in adults aged 20–74 years [1]. With the increasing prevalence of diabetes, the number of instances of DR and vision-threatening DR (VTDR), has been estimated to rise to 191.0 million and 56.3 million, respectively, by 2030 [2]. DR is a complex trait involving polygenic, metabolic, and environmental influence. Known risk factors—most notably duration of diabetes and glycemic control—explain some, but not all, of the progression of DR [3,4,5]. There are diabetic patients that develop DR despite short durations of diabetes and/or excellent glycemic control, and other diabetic patients who do not develop DR in the face of long-standing diabetes and/or long-term hyperglycemia [6]. Therefore, given the complexity of the disease, genetic factors may explain some of the variation in the development of DR [7].

The gene encoding methylenetetrahydrofolate reductase (MTHFR, chromosome 1p36.3), which catalyzes the methylation of homocysteine to methionine [8], has been widely regarded as a genetic candidate for diabetes mellitus (DM). It has been demonstrated that the single nucleotide polymorphism (SNP) in *MTHFR* gene at nucleotide 677C/T(amino acid 222Ala/Val) can destroy the enzyme activity and cause hyperhomocysteinemia (HHcy) [9,10,11]. Because of such critical functional influence, it is readily postulated that the polymorphism of *MTHFR* 677C/T contributes to the development of DR, and a number of studies have addressed the role of the variation in the complex etiology of DR.

Numerous molecular epidemiological studies have been performed to estimate the relationship between the *MTHFR* 677C/T polymorphism and DR [12,13,14,15,16,17,18,19,20,21,22,23,24,25,26,27,28,29], but the results remain inconclusive. Although several meta-analyses have been published [30,31], they still did not reach a consistent conclusion. To better shed light on these conflicting findings, we conducted a comprehensive meta-analysis on 18 published studies from 1996 to 2016, with 1747 diabetic retinopathy cases and 3146 controls relating the variant of the *MTHFR* 677C/T to the risk of developing DR.

## 2. Methods

This study was reported according to the Meta-analysis of Observational Studies in Epidemiology (MOOSE) guidelines and Preferred Reporting Items for Systematic Reviews and Meta-Analyses (PRISMA) for reporting systematic reviews and meta-analyses. Study selection, data extraction, and quality assessment were completed independently by two investigators. Disagreement was resolved through discussion. If the discussion did not lead to a consensus, Professor Wang made the final decision.

### 2.1. Identification and Eligibility of Relevant Studies

We attempted to include all the studies that determined the genotype distribution of *MTHFR* 677C/T polymorphism in cases of diabetes retinopathy, and (i) in healthy controls or (ii) in diseased controls (subjects with diabetes and free of DR) in the meta-analysis. Cases were type 1 or 2 diabetic subjects with background, simple, advanced, or proliferative DR. The diseased control group consisted of subjects with diabetes and free of diabetic retinopathy disease, i.e., diabetes nephropathy.

We first identified studies by searching the electronic literature PubMed and Embase for relevant reports in English and CNKI for papers in Chinese (from January 1996 to April 2016, using the search terms “(MTHFR or methylenetetrahydrofolate reductase) and (diabetes or diabetic) and (retinopathy) and (gene or polymorphism or allele or genotype or variant or variation or mutation)” [30]. We chose articles which were conducted among human subjects. Eligible studies were then identified by further searching the studies on the association between *MTHFR* 677C/T polymorphism and diabetic retinopathy risk. We restricted attention to the studies that satisfied all of the following criteria: (1) studies related to the *MTHFR* polymorphism were determined regardless of sample size and study design (case-control, cross-sectional or cohort studies); (2) each genotype frequency was reported; and (3) there was sufficient information for extraction of data. If studies had partly overlapped subjects, only the one with a larger and/or latest sample size was selected for the analysis. Additional studies were identified by hands-on searches from references of original studies or review articles on this topic. According to these criteria, we finally included 18 papers in our meta-analysis.

### 2.2. Data Extraction and Conversion

Two investigators independently extracted data and reached a consensus on all of the items. Data extracted from these articles included the first author’s name, publication year, study design, ethnicity of population, DM type, clinical characteristics, and the number of cases and controls for *MTHFR* C677T genotypes. The frequencies of the alleles and the genotypic distributions were extracted or calculated for both cases and controls. We merged the original data into the control group or case group if the study did not provide corresponding data. For some studies without sufficient information for extraction of data, we tried to contact with the studies’ authors by sending emails from their articles to request missing data. In addition, it was tested whether the distribution of genotypes in the controls was consistent with Hardy–Weinberg equilibrium (HWE) for each study, and the frequency of the minor allele for *MTHFR* 677C/T polymorphism was calculated.

### 2.3. Quality Assessment and Study Stratification

We used the Newcastle–Ottawa scale (NOS) method to assess the observational studies that were included. The NOS is composed of 3 parts (8 entries): selection, comparability, and exposure. A quality item is given only one star for the study in selection and exposure, and a quality item is given at most two stars for the study in comparability. It is a semi-quantitative scale, and a score of 0–9 stars is assigned to each study. Studies whose scores were more than 6 stars were considered to be of relatively high quality [32]. The scores of included studies are shown in Table 1.

### 2.4. Meta-Analysis

Our meta-analysis evaluated the relationship between the *MTHFR* 677C/T polymorphism and the risk of DR for each study by odds ratio (OR) with 95% confidence intervals (95% CI). For all studies, we calculated the ORs for the: (i) separate pairwise comparisons; (ii) allele contrast; (iii) recessive model; and (iv) dominant model. In addition, we conducted stratification analysis by ethnicity and DM type. A sensitivity analysis, which examines the effect of excluding specific studies, was also performed [33]. Our meta-analysis was subjected to sensitivity analysis for studies with the controls not in HWE (*p* < 0.05).

The χ^2^-based Q statistic test was used to assess the heterogeneity, and it was considered significant for *p* < 0.05. Heterogeneity was quantified with the *I*^2^ metric, which is independent of the number of studies in the meta-analysis. *I*^2^ takes values between 0% and 100%, with higher values denoting greater degree of heterogeneity (*I*^2^ > 50% was considered significant) [34]. We used the fixed-effects model and the random-effects model based on the Mantel–Haenszel method and the DerSimonian and Laird method, respectively, to combine values from each of the studies. When the effects were assumed to be heterogeneous, the random-effects model was used; otherwise, the fix-effects model was more appropriate [35]. In addition, we further conducted meta-regression analyses to estimate the source of heterogeneity. Publication bias was assessed according to the Begg adjusted rank correlation test and the Egger regression asymmetry test [36,37]. All analysis was done using the Stata software (v.12.1) (StataCorp LP, College Station, TX, USA). All the *p* values were two-sided.

## 3. Results

### 3.1. Literature Search

**Figure 1:**
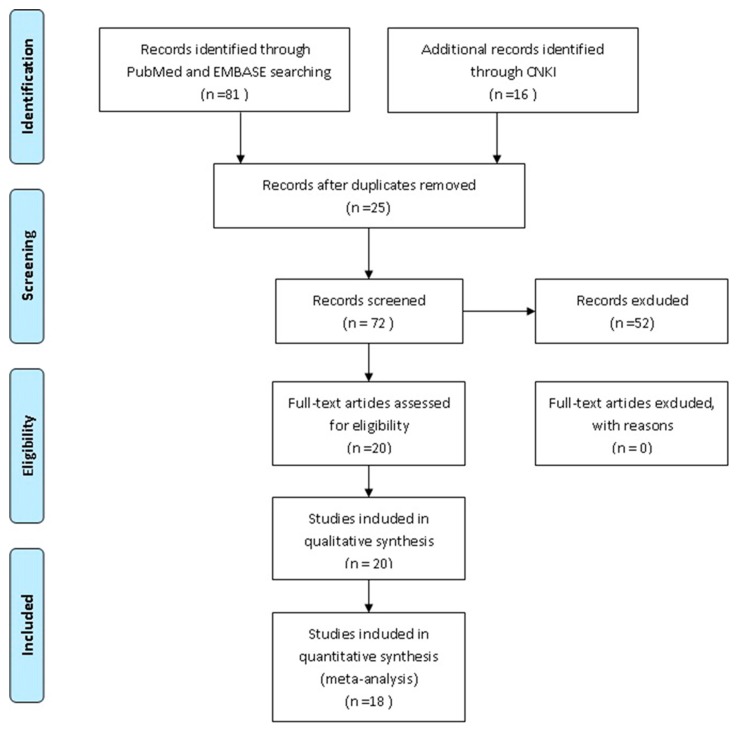
Flow chart of the literature search.

The study selection process is shown in Figure 1. A total of 97 articles (PubMed 35, Embase 46 and CNKI 16) were identified from the databases, and 25 duplicates were excluded using EndNote (X7). In addition, 52 articles were excluded based on a review of the titles and abstracts, and 20 full-text articles were assessed for eligibility; 2 articles were excluded due to could not provide each genotype frequency or other sufficient information for extraction of data. Finally, a total of 18 [12,13,14,15,16,17,18,19,20,21,22,23,24,25,26,27,28,29] articles were included in this meta-analysis.

### 3.2. Eligible Studies and Study Characteristics

The selected study baseline characteristics from the qualified studies included in the meta-analysis are provided in Table 1 and the frequencies on *MTHFR* C677T polymorphism allele/genotype prevalence are shown in Table 2. Of 18 studies, 12 studies (9 Asian, 2 Caucasian, and 1 American population) were based on type 2 DM (T2DM) including participants (case group; control group), and 6 studies (3 Caucasian and 3 Asian) was based onnon-T2DM (2 studies ([12,14]) with type 1 DM (T1DM), 2 ([21,24]) with un-defined DM type, and 2 ([23,28]) involved both T1DM and T2DM) including participants (case group; control group). Ten studies were case-control study design and 8 studies were cross-sectional study design.

### 3.3. Summary Statistics

Data from 18 articles that investigated the association between the *MTHFR* 677C/T polymorphism and DR risk were included in the meta-analysis. The overall frequency (%) of minor D allele frequency (MAF) was 0.44/0.33 for cases and controls. The frequency of the MAF for each individual study polymorphism for controls is given in Table 1. All studies indicated that the distribution of genotypes in the controls was consistent with Hardy–Weinberg equilibrium except for 3 studies ([19,22,24]), indicating genotyping errors and/or population stratification [33]; therefore, a sensitivity analysis was performed by excluding these studies.

### 3.4. Main Results, Stratification, and Sensitivity Analyses

**Figure 2:**
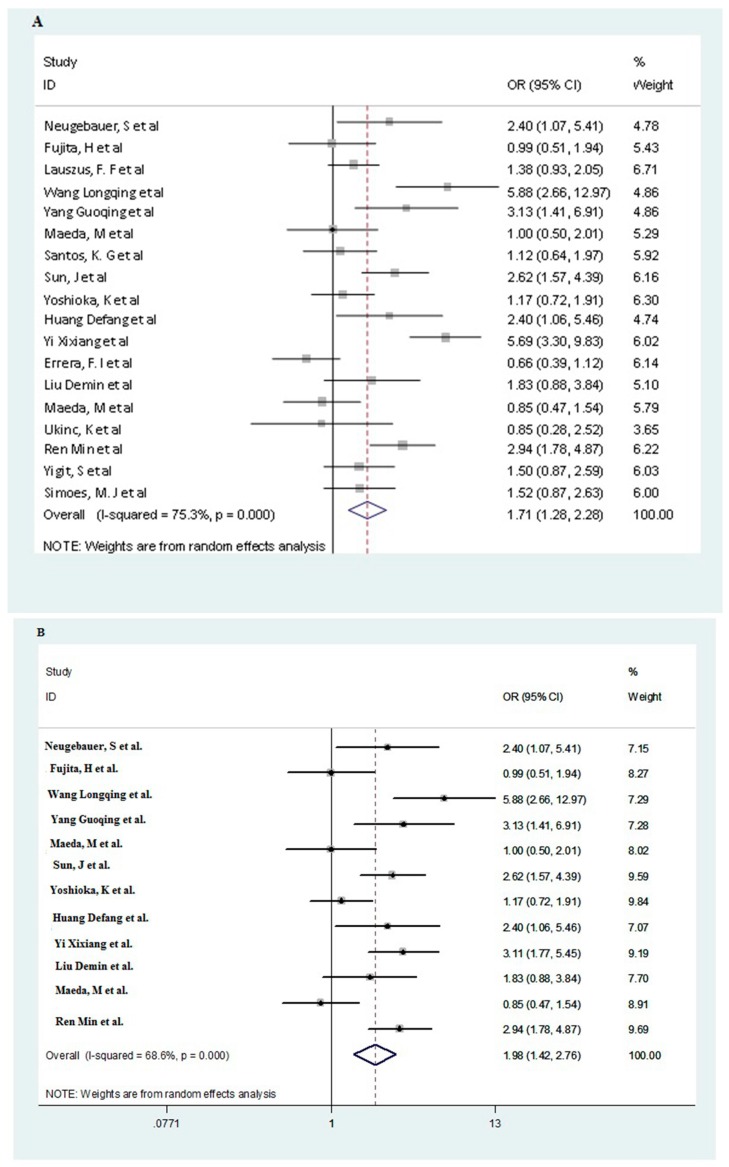

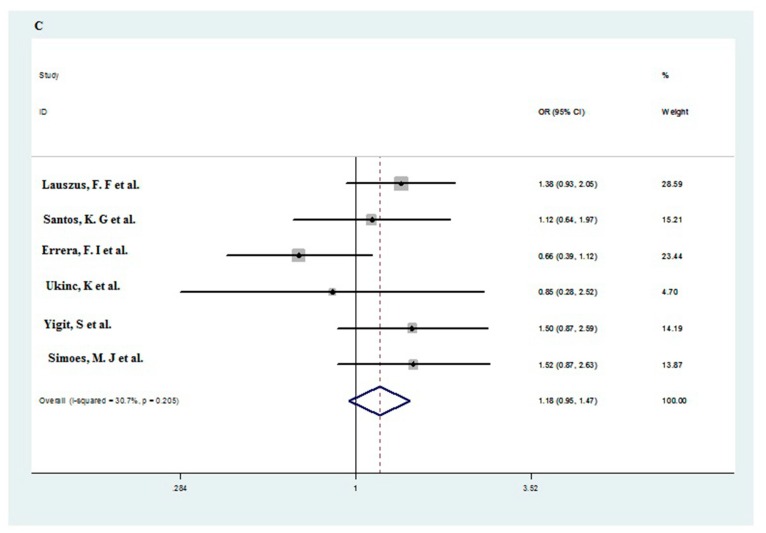
Oddsratios (ORs; log scale) of diabetic retinopathy (DR) associated with *MTHFR* 677C/T polymorphism for dominant genetic model. The graph shows individual and pooled estimates for different ethnic groups (**A** for all studies; **B** for Asian group; **C** for Non-Asian group).

The estimation results of the relationship of *MTHFR* 677C/T polymorphism with DR are presented in Table 3. Figure 2 shows the overall results for the association between the polymorphism and the risk of DR (in dominant model).

As it shown in Table 3, the overall analysis found a significant association between the *MTHFR* 677C/T polymorphism and the risk of DR for all genetic models (CT vs. CC: OR = 1.46, 95% CI: 1.15–1.86; TT vs. CC:OR = 2.45, 95% CI: 1.66–3.60; Allele contrast: OR = 1.52, 95% CI: 1.26–1.84; recessive model: OR = 1.67, 95% CI: 1.19–2.40 and dominant model: OR = 1.71, 95% CI: 1.28–2.28, respectively).

In stratified analysis by ethnicity and DM type, we further detected that the Asian group, T2DM group showed significant associations for all genetic models (CT vs. CC: OR = 1.71, 95% CI: 1.22–2.39 for Asian group, OR = 1.50, 95% CI: 1.08–2.09 for T2DM group, respectively; TT vs. CC:OR = 2.97, 95% CI: 2.06–4.29 for Asian group, OR = 2.68, 95% CI: 1.74–4.13 for T2DM group, respectively; Allele contrast: OR = 1.75, 95% CI: 1.42–2.18 for Asian group, OR = 1.59, 95% CI: 1.26–2.01 for T2DM group, respectively; recessive model: OR = 2.16, 95% CI: 1.75–2.65 for Asian group, OR = 2.05, 95% CI: 1.47–2.84 for T2DM group, respectively and dominant model: OR = 1.98, 95% CI: 1.42–2.76 for Asian group, OR = 1.72, 95% CI: 1.23–2.42 for T2DM group, respectively). In addition, we further found significant associations for all genetic models in both Asian group with T2DM and non-T2DM (See Table 3). However, we did not find any significant effects for different genetic models in other subgroups. Further sensitivity analysis for HWE almost did not alter the pattern of results in both overall analysis and subgroup analysis (See Table 3).

### 3.5. Source of Heterogeneity and Publication Bias

From Table 3, we found that the heterogeneity between studies was observed in overall comparisons as well as subgroup analyses. We estimated the source of heterogeneity in dominant genetic models of the variant allele by ethnicity (Asian or non-Asian), DM type (T2DM or non-T2DM), HWE (in HWE or not), and study design (case-control or cross-sectional design) by meta-regression analyses. It revealed that DM type and HWE factors could not significantly influence between-study heterogeneity in genetic model for the polymorphism *MTHFR* 677C/T: DM type (*p* = 0.36) and HWE (*p* = 0.06). However, we found that ethnicity and study design factors might be the source of heterogeneity between studies: ethnicity (*p* = 0.03 and contributed 26.8% source of heterogeneity) and study design (*p* = 0.01 and contributed 52.1% source of heterogeneity). In addition, we further found that study design might be the major source of heterogeneity between studies for the Asian DM group (*p* = 0.001 and contributed 100% source of heterogeneity).

**Figure 3:**
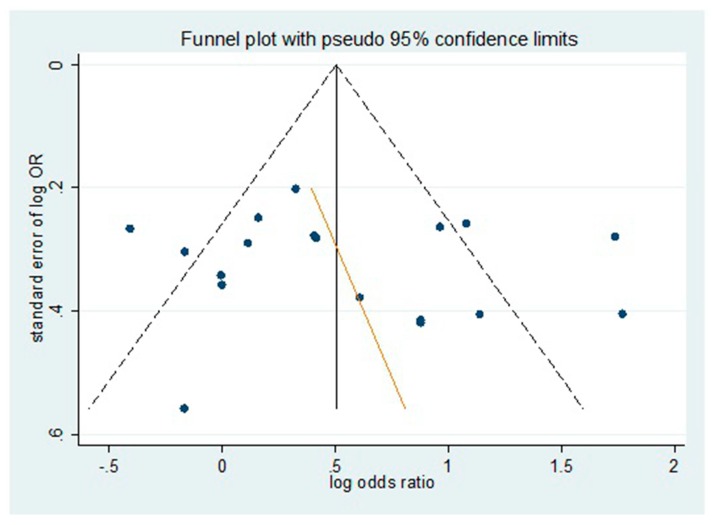
Evaluation of publication bias for all studies using funnel plots. No significant funnel asymmetry was observed which could indicate publication bias.

The potential presence of publication bias was estimated by using a funnel plot by evaluating log–odds ratio for the genotype TT + CT versus CC against the reciprocal of its standard error (Figure 3). As is shown, we failed to observe any significant funnel asymmetry which could indicate publication bias. We further conducted the Egger regression asymmetry test and the Begg adjusted rank correlation tests to estimate the publication bias of included literatures in the meta-analysis. As shown in Table 4, no publication bias was found for the polymorphism and risk of DR in both dominant and recessive genetic models.

## 4. Discussion

The common environmental risk factors of DR include hyperglycemia, hypertension, hyperlipidemia, obesity, duration of diabetes, puberty, and pregnancy [2]. However, despite having a long-term hyperglycemia, we often found that some diabetics develop retinopathy, whereas others do not. Because such known environmental factors do not fully explain this, researchers have sought the answer in genetic factors. The enzyme MTHFR methylates homocysteine to generate methionine, and its dysfunction can lead to HHcy [38]. Studies reported that HHcy induces endothelial dysfunction and arterial stiffness [39], and has also been associated with atherosclerosis [40] and retinopathy in both T1DM and T2DM patients [41,42]. The *MTHFR* C677T polymorphism leads to an Ala222Val substitution in the N-terminal catalytic domain of the enzyme, which reduces enzyme activity [43,44,45]. In addition, several recent genome-wide association studies (GWAS) have confirmed the association between *MTHFR* C677T genotype and homocysteine levels in healthy populations [46,47]. Numerous investigations into the potential role of *MTHFR* as a susceptibility gene for DR have been conducted over the past decades, with controversial results. Early meta-analyses attempted to reconcile these findings, but attempts to draw definite conclusions have been hindered by limited data, particularly when examining specific patient subgroups and increased relative studies [30,31].

In the current meta-analysis, the effect of separate pairwise comparisons, allele contrast, and the effects of the dominant and recessive genetic models were estimated. Subgroup analysis by ethnicity and DM type, and sensitivity for studies not in HWE, were performed. In addition, we further evaluated the source of heterogeneity and publication bias of included literatures. It is worth mentioning that we found study design might be the major source of heterogeneity between studies. To provide better power to detect smaller effect sizes, studies related to the *MTHFR* polymorphism were determined regardless of sample size and study design (case-control, cross-sectional, or cohort studies), however, different study designs may generate potential effects on the meta-analysis. For example, the cohort study design may obtain a much more powerful research conclusion than the case-control study design, and the cross-sectional study design could obtain less powerful research conclusion than the case-control study design. Therefore, the overall effect of all studies with different study designs might be deviated from the real effect, to some extent.

Our meta-analysis obtained several critical different conclusions from the previous reports [30,31]. In Zintzaras et al. [31] report, they just included 5 studies with 435 DR cases and 620 controls, which provides relatively poor power to detect smaller effect sizes. In addition, although they found a marginal association between C677T and the risk of developing DR, the results of overall analysis were less significant after conducting sensitivity analysis by excluding the study with the control not in HWE. Niu et al. [30] just performed the meta-analysis with 8 studies including 1599 subjects and they did not observe significant association with DR in heterozygous genotypic comparison (CT vs. CC) or in dominant model. In addition, they did not perform meta-regression analysis to identify the sources of between-study heterogeneity and found publication bias for comparison. However, from the present meta-analysis of 18 studies—reported from 1996 to 2016 and comprising 4893 subjects—we not only found the main effects for *MTHFR* C677T polymorphism on DR risk with all studies in all genetic models, without any publication bias, but further sensitivity analysis for HWE also did not alter the pattern of results in the overall analysis. From the stratification analysis by ethnicity and DM type, we also found that the *MTHFR* C677T polymorphism was significantly associated with DR risk in T2DM and Asian group, especially in Asian group with T2DM and non-T2DM.These findings may indicate that genetic factors may have more impact on Asian population.

We conducted a comprehensive meta-analysis on 18 published studies with 1747 DR cases and 3146 controls relating to the mutation of the *MTHFR* C677T to the risk of DR, which can provide better power to detect smaller effect sizes. Its strength was based on the accumulation of published data giving greater information to detect significant differences. In order to estimate the power of the study, we used the Power and Precision 4 software to conduct the power calculation by respectively accumulating the frequency of *MTHFR* 677T allele in case (0.44) and control (0.33) groups from all studies, and the result showed the power of our study is over 80%.

Despite the clear strengths of our study, some limitations merit serious consideration. First, non-English/Chinese, non-indexed, and non-published literature were not reviewed in our meta-analysis, thus might introduce some bias [48]. Second, only the unadjusted pooled ORs were calculated, because data for possible confounding factors that influence the estimates of associations (e.g., age, sex, body mass index) were not provided. Third, sampling variability and stratification in genetic association studies could be a possible confounding factor on the role of genetic markers. In addition, the risk effect may depend on the interaction with other risk factors: diabetes duration, HbA_1c_, blood pressure, total serum cholesterol, control of diabetes, and body mass index, all of which modulate the development of DR [49]. Furthermore, small numbers of individuals and inadequate information of lifestyle factors and dietary intake by the published studies limited our statistic power to fully investigate the gene-environment interactions [35]. Therefore, further well-designed large studies, particularly referring to GWAS and gene-environment interactions are warranted to confirm the real contribution of these polymorphisms to DR susceptibility and might further elucidate the genetics of DR.

Although there are several previous GWAS relevant to DR [50,51,52,53], the limitations in GWAS studies are still inconsistency and low reproducibility in different populations [2]. Several reasons may explain this [7]: (i) since the genetic effects of DR might be much modest than other diseases, it requires large sample sizes to identify the real role of genetic factors using GWAS, however, the sample sizes of previous studies on DR GWAS studies were relatively modest; (ii) the diagnosis of DR is clinically complicated with different types of DM, a case with slight retinopathic changes may not be assigned as a DR case, but some of the DR GWAS used case definitions that include such patients which may generate the bias. In addition, different population and heterogeneous phenotypes of DR patients, as well as poor characterization of controls, also affect the consistency of GWAS studies. Therefore, it is still very important to conduct the meta-analysis to estimate the variant of the *MTHFR* C677T to the risk of DR.

## 5. Conclusions

In summary, our present meta-analysis finds a relationship between DR and *MTHFR* C677T polymorphism, especially in Asian groups. Prospective and additional GWAS are needed to clarify the real role of the *MTHFR* gene in the development of DR.

## Figures and Tables

**Table 1:** Baseline characteristics of qualified studies included in the meta-analysis.

Author (Ref *)	Year	Ethnicity	Design	Case				Control						
				Sample Size	Age (year)	DM Duration (year)	Definition	Sample Size	Age (year)	DM Duration (year)	Definition	HWE ^#^	MAF ^&^	NOS (Stars)
Neugebauer, S. et al. [12]	1997	Japan	CS	76	55.5 ± 7.9	16.5 ± 5.1	DR	36	50.5 ± 9.7	11.2 ± 4.2	N*CT*DM	0.67	0.26	7
Fujita, H. et al. [13]	1999	Japan	CS	105	60±12	NR	PDR + DN	68	62 ± 10	NR	T2DM	0.14	0.42	6
Lauszus, F.F. et al. [14]	2001	Denmark	CS	112	NR	NR	DR	1084	NR	NR	T1DM	0.53	0.29	5
Wang, L.Q. et al. [15]	2001	China	CC	62	62.50 ± 8.08	8.29 ± 6.40	DR	202	59.42 ± 14.87 for T2DM41.83 ± 17.10 for Healthy	7.29 ± 5.80 for T2DM	Healthy + T2DM	0.73	0.32	8
Yang, G.Q. et al. [16]	2001	China	CC	60	50.7 ± 12.1	1 (0.1–4)	DR	231	51.1 ± 12.8 for T2DM with DN63.0 ± 8.8 for T2DM with NCD52.6 ± 14.9 for Healthy	2 (1–4) for T2DM with DN14 (11–18) for T2DM with NCD	Healthy + T2DM	0.73	0.44	8
Maeda, M. et al. [17]	2003	Japan	CS	51	NR	NR	DR	105	NR	NR	T2DM	0.06	0.37	5
Santos, K.G. et al. [18]	2003	Brazil	CS	99	NA	NA	DR	111	NA	NA	T2DM	0.98	0.39	6
Sun, J. et al. [19]	2003	China	CC	110	55.6 ± 6.7	<5	DR	155	54.7 ± 7.1 for NDR42.3 ± 6.1 for Healthy	>10 for NDR	T2DM	0.00	0.33	7
Yoshioka, K. et al. [20]	2003	Japan	CS	98	NA	NA	DR	268	NA	NA	T2DM	0.46	0.38	6
Huang, D.F. et al. [21]	2005	China	CC	50	NR	NR	DR	47	NR	NR	Healthy	0.96	0.26	5
Yi, X.X. et al. [22]	2005	China	CC	249	56.53 ± 10.45	5.9 ± 4	DR	65	NR	NR	Healthy	0.01	0.31	5
Errera, F.I. et al. [23]	2006	Brazil	CC	141	55.43 ± 15.33	18 ± 8.67	DR	107	66.11 ± 7.06	NA	Healthy	0.24	0.40	6
Liu, D.M. et al. [24]	2006	China	CC	44	51.9 ± 7.5	NR	DR	84	54.0 ± 13.2	NA	Healthy	0.01	0.29	5
Maeda, M. et al. [25]	2008	Japan	CS	75	NA	NA	DR	115	NA	NA	T2DM	0.06	0.36	5
Ukinc, K. et al. [26]	2009	Turkey/	CS	25	NA	NA	DR	27	NA	NA	T2DM	0.10	0.24	5
Ren, M. et al. [27]	2011	China	CC	161	59.95 ± 10.55	11	DR	213	58.52 ± 12.61	7	T2DM	0.23	0.42	7
Yigit, S. et al. [28]	2013	Turkey	CS	81	NA	NA	DR	149	NA	NA	T1DM + T2DM	0.98	0.24	5
Simoes, M.J. et al. [29]	2014	Portugal	CS	148	58.5 ± 8.4	10.5 ± 5.3	PDR	79	61.8 ± 7.8	8.9 ± 4.8	T2DM	0.73	0.24	7

* The ref was referred to the reference numbers in this study; ^#^ Hardy–Weinberg equilibrium (HWE) test and ^&^ the minor allele frequency (MAF) were calculated in control group for each study. NR, not reported; NA, not available; CC, case-control; CS, cross-sectional; CH cohort; DR, diabetes retinopathy; PDR, proliferative diabetes retinopathy; NCD: non-complicated; DN, diabetes nephropathy;T1DM, type 1 diabetes mellitus; T2DM, type 2 diabetes mellitus; N*CT*DM, non-insulin dependent diabetes mellitus.

**Table 2:** The frequency of methylenetetrahydrofolate reductase gene (*MTHFR*) C677T polymorphism allele/genotype prevalence.

Author (Ref)	Prevalence of MTHFR C677T Genotype						Prevalence of Allele Frequency			
	CC		CT		TT		C		T	
	Case	Control	Case	Control	Case	Control	Case	Control	Case	Control
Neugebauer, S. et al. [12]	26	20	38	13	12	3	90	53	62	19
Fujita, H. et al. [13]	31	20	57	39	17	9	119	79	91	57
Lauszus, F.F. et al. [14]	47	542	57	455	8	87	151	1539	73	629
Wang, L.Q. et al. [15]	8	94	27	86	27	22	43	274	81	130
Yang, G.Q. et al. [16]	8	75	33	111	19	45	49	261	71	201
Maeda, M. et al. [17]	18	37	20	58	13	10	56	132	46	78
Santos, K.G. et al. [18]	34	41	53	53	12	17	121	135	77	87
Sun, J. et al. [19]	33	82	46	45	31	28	112	209	108	101
Yoshioka, K. et al. [20]	33	100	50	132	15	36	116	332	80	204
Huang, D.F. et al. [21]	17	26	25	18	8	3	59	70	41	24
Yi, X.X. et al. [22]	68	35	110	19	71	11	246	89	252	41
Errera, F.I. et al. [23]	61	36	66	57	14	14	188	129	94	85
Liu, D.M. et al. [24]	18	47	16	25	10	12	52	119	36	49
Maeda, M. et al. [25]	31	43	28	62	16	10	90	148	60	82
Ukinc, K. et al. [26]	14	14	11	13	0	0	39	41	11	13
Ren, M. et al. [27]	26	77	78	95	57	41	130	249	192	177
Yigit, S. et al. [28]	38	85	30	55	13	9	106	225	56	73
Simoes, M.J. et al. [29]	69	45	60	30	19	4	198	120	98	38
Total	580	1419	805	1366	362	361	1965	4204	1529	2088

**Table 3:** Summary ORs and heterogeneity results for associations between the *MTHFR* C677T polymorphism and DR.

Subgroup	Genetic Model	Studies No (All/Sensitivity)	OR (95% CI)	*p* *	*I*^2^ (%)	OR _se_ (95% CI) ^#^
Overall	CT vs. CC	18/15	1.46 (1.15–1.86)	0.00	60.0	1.32 (1.02–1.70)
	TT vs. CC	18/15	2.45 (1.66–3.60)	0.00	69.0	2.27 (1.44–3.58)
	Allele contrast	18/15	1.52 (1.26–1.84)	0.00	72.4	1.45 (1.17–1.79)
	Recessive model	18/15	1.67 (1.19–2.40)	0.00	71.9	1.87 (1.32–2.66)
	Dominant model	18/15	1.71 (1.28–2.28)	0.00	75.3	1.50 (1.13–1.98)
Asian	CT vs. CC	12/9	1.71 (1.22–2.39)	0.00	64.9	1.52 (1.01–2.30)
	TT vs. CC	12/9	2.97 (2.06–4.29)	0.02	51.5	3.07 (1.83–5.14)
	Allele contrast	12/9	1.75 (1.42–2.18)	0.00	67.3	1.69 (1.28–2.24)
	Recessive model	12/9	2.16 (1.75–2.65)	0.11	35.1	2.34(1.62–3.38)
	Dominant model	12/9	1.98 (1.42–2.76)	0.00	68.6	1.83 (1.20–2.81)
Non-Asian	CT vs. CC	6/6	1.15 (0.92–1.45)	0.38	5.4	1.15 (0.92–1.45)
	TT vs. CC	6/6	1.33 (0.69–2.54)	0.04	61.4	1.33 (0.69–2.54)
	Allele contrast	6/6	1.14 (0.89–1.46)	0.07	51.5	1.14 (0.89–1.46)
	Recessive model	6/6	1.24 (0.69–2.23)	0.05	57.7	1.24 (0.69–2.23)
	Dominant model	6/6	1.18 (0.95–1.47)	0.21	30.70	1.18 (0.95–1.47)
T2DM	CT vs. CC	12/10	1.50 (1.08–2.09)	0.00	66.5	1.32 (0.93–1.86)
	TT vs. CC	12/10	2.68 (1.74–4.13)	0.00	66.2	2.61 (1.50–4.53)
	Allele contrast	12/10	1.59 (1.26–2.01)	0.00	74.0	1.49 (1.14–1.96)
	Recessive model	12/10	2.05 (1.47–2.84)	0.01	56.9	2.10 (1.38–3.17)
	Dominant model	12/10	1.72 (1.23–2.42)	0.00	72.1	1.54 (1.06–2.24)
Non-T2DM	CT vs. CC	6/5	1.30 (1.02–1.66)	0.12	43.5	1.32 (0.89–1.97)
	TT vs. CC	6/5	1.75 (0.93–3.27)	0.05	55.9	1.70 (0.80–3.63)
	Allele contrast	6/5	1.38 (1.02–1.88)	0.02	64.5	1.34 (0.95–1.91)
	Recessive model	6/5	1.38 (0.96–2.00)	0.14	39.5	1.32 (0.89–1.97)
	Dominant model	6/5	1.46 (1.00–2.13)	0.04	57.4	1.42 (0.92–2.19)
Asian with T2DM	CT vs. CC	9/7	1.64 (1.07–2.50)	0.00	73.8	1.40 (0.86–2.29)
	TT vs. CC	9/7	3.00 (1.92–4.68)	0.01	63.8	2.99 (1.63–5.50)
	Allele contrast	9/7	1.73 (1.33–2.26)	0.00	76.0	1.64 (1.17–2.29)
	Recessive model	9/7	2.21 (1.59–3.06)	0.03	51.9	2.33 (1.52–3.57)
	Dominant model	9/7	1.94 (1.27–2.94)	0.00	76.7	1.72 (1.03–2.89)
Asian with Non-T2DM	CT vs. CC	3/2	1.99 (1.22–3.25)	0.87	0.0	2.19 (1.19–4.01)
	TT vs. CC	3/2	2.80 (1.39–5.66)	0.77	0.0	3.50 (1.28–9.62)
	Allele contrast	3/2	1.86 (1.32–2.60)	0.90	0.0	1.97 (1.28–3.05)
	Recessive model	3/2	2.06 (1.06–4.02)	0.87	0.0	2.38 (0.91–6.24)
	Dominant model	3/2	2.17 (1.38–3.42)	0.85	0.0	2.40 (1.35–4.28)
Non-Asian with T2DM	CT vs. CC	3/3	1.20 (0.81–1.76)	0.79	0.0	1.20 (0.81–1.76)
	TT vs. CC	3/3	1.54 (0.43–5.46)	0.08	68.2	1.54 (0.43–5.46)
	Allele contrast	3/3	1.18 (0.90–1.56)	0.25	27.0	1.18 (0.90–1.56)
	Recessive model	3/3	1.37 (0.39–4.82)	0.06	70.8	1.37 (0.39–4.82)
	Dominant model	3/3	1.24 (0.86–1.80)	0.58	0.0	1.24 (0.86–1.80)
Non-Asian with Non-T2DM	CT vs. CC	3/3	1.09 (0.69–1.71)	0.09	58.0	1.09 (0.69–1.71)
	TT vs. CC	3/3	1.24 (0.49–3.14)	0.03	71.9	1.24 (0.49–3.14)
	Allele contrast	3/3	1.13 (0.76–1.68)	0.02	73.3	1.13 (0.76–1.68)
	Recessive model	3/3	1.21 (0.54–2.72)	0.05	66.9	1.21 (0.54–2.72)
	Dominant model	3/3	1.13 (0.69–1.83)	0.05	66.7	1.13 (0.69–1.83)

^#^ OR _se_: Sensitivity analysis OR for HWE; * Test for heterogeneity: random-effects model was used when *p* value for heterogeneity test <0.05 and *I*^2^ > 50%; otherwise, fixed-effects model was used; T2DM, type 2 diabetes mellitus;Non-T2DM, type 1 diabetes mellitus or unknown DM type or mixed DM type; Non-Asian, Caucasian and African-American population.

**Table 4:** The results of publication bias test by Egger and Begg test.

Subgroup	Egger Test		Begg Test	
	Dominant	Recessive	Dominant	Recessive
all study	0.59	0.48	0.65	0.48
T2DM	0.91	0.94	0.84	0.64
Non-T2DM	0.28	0.16	0.06	0.26
Asian	0.62	0.91	0.63	0.54
Non-Asian	0.57	0.10	1.00	0.46

T2DM, type 2 diabetes mellitus;Non-T2DM, type 1 diabetes mellitus or unknown DM type or mixed DM type; Non-Asian, Caucasian and African-American population.
